# Deux Oeufs*:* cracking the potential of eggs to improve child growth and development — a randomized controlled trial (RCT) study protocol

**DOI:** 10.1186/s13063-025-09237-3

**Published:** 2025-12-23

**Authors:** Chhavi Tiwari, Daniel Acosta, Eric Matsiko, Apolline Kampire, Aloys Nsabimana, Heather Stark, Juan E. Andrade Laborde, Yang Yang, Kathryn Reider, Etienne Nsereko, Miles Kirby, Sarah McKune

**Affiliations:** 1 Department of Environmental and Global Health, University of Florida, Gainesville, USA; 2 College of Medicine and Health Sciences, University of Rwanda, Kigali, Rwanda; 3 Health and Nutrition, World Vision Rwanda, Kigali, Rwanda; 4 Department of Epidemiology, University of Florida, Gainesville, USA; 5 Department of Food Science and Human Nutrition, University of Florida, Gainesville, USA; 6 Department of Statistics, Franklin College of Arts and Sciences, University of Georgia, Atlanta, USA; 7 International Programs Group, World Vision US, Washington D.C., USA

**Keywords:** Pregnancy, Eggs, Animal source foods, Randomized controlled trial, Nutrition

## Abstract

**Background:**

The first thousand days post-conception are crucial for potential interventions to improve early child growth and development. Adequate nutrition during pregnancy plays a pivotal role in both maternal and child health. Recent studies indicate that the consumption of animal-source foods (ASF) such as eggs during childhood may have a positive effect on child growth and development. However, limited evidence exists for the impact of egg supplementation during pregnancy on maternal and child nutrition. The *Deux Oeufs* study aims to test the effect of a maternal nutritional intervention of two eggs per day during pregnancy on birth outcomes and infant growth.

**Methods:**

*Deux Oeufs* is a randomized controlled trial (RCT) and will aim to enroll and randomize 956 pregnant women from *Nyagatare* District in Rwanda into one of two study arms: a treatment group (T1) or a control group (T2). Women in the treatment arm will consume a supplement of two eggs per day starting from enrollment during the first trimester of pregnancy until childbirth. The study staff will directly observe the consumption of eggs daily. Women in the control arm will continue their usual diet. All enrolled participants will receive standard prenatal care, as outlined by the Government of Rwanda (GoR), a mobile phone, health insurance (if not already covered), and modest compensation for participation. All participants who are not already receiving Shisha Kibondo (a fortified flour supplement provided by GoR) as part of their standard care will receive the same amount (6 kg/month) from the study, regardless of treatment arm. The primary outcome is child length-for-age z-score (LAZ) at birth. Secondary outcomes include maternal health indicators (during and after pregnancy) and child growth and early development measures both in utero, during the second and third trimesters, after birth, and at 6 weeks and 6 months of age. Women will be enrolled on a rolling basis starting from May 2024.

**Discussion:**

*Deux Oeufs* is the first RCT to test the effects of directly observed daily maternal egg consumption during pregnancy on child nutrition outcomes. Findings from the study will provide evidence on the potential for prenatal ASF supplementation to improve child growth and development and consequently inform policy decisions.

**Trial registration:**

ClinicalTrials.gov NCT06405360. Registered on May 03, 2024.

## Administrative information

**Table:** 

Title {1}	Deux Oeufs*:* cracking the potential of eggs to improve child growth and development — a randomized controlled trial study protocol
Trial registration {2a and 2b}	ClinicalTrials.gov identifier: NCT06405360 Date registered: 03 May 2024
Protocol version {3}	Version 2, 24 October 2024
Funding {4}	This work was funded in whole or part by the United States Agency for International Development (USAID) Bureau for Resilience, Environment and Food Security under Agreement no. AID-OAA-L-15-00003 as part of Feed the Future Innovation Lab for Livestock Systems. Additional funding was received from Bill & Melinda Gates Foundation OPP no. 060115. Any opinions, findings, conclusions, or recommendations expressed here are those of the authors alone
Author details {5a}	Chhavi Tiwari^1^, Daniel Acosta^1^, Eric Matsiko^2^ Apolline Kampire^2^, Aloys Nsabimana^3^, Heather Stark^4^, Juan E. Andrade Laborde^5^, Yang Yang^6^, Kathryn Reider^7^, Etienne Nsereko^2^, Miles Kirby^7^, Sarah McKune^1^
Name and contact information for the trial sponsor {5b}	Sarah McKune, University of Florida, smckune@ufl.edu
Role of sponsor {5c}	The role of USAID was limited to funding. Any opinions, findings, conclusions, or recommendations expressed here are those of the authors alone. The USAID funding was allocated for a period of three years from October 01, 2022, to September 30, 2025. USAID funding was suspended on January 27, 2025; efforts are ongoing without support or use of USAID funds
Roles and responsibilities: committees {5d}	The project is led by Dr. Sarah McKune (University of Florida, PI), with scientific leadership contributions from Dr. Juan Andrade (Co-PI), Dr. Heather Stark (Co-PI), and Dr. Yang Yang (Co-PI). Dr. Etienne Nsereko and Dr. Eric Matsiko will serve as scientific lead from University of Rwanda. World Vision US serve as subawardee with Dr. Miles Kirby as subaward PI. World Vision Rwanda will be responsible for the implementation of the trial

## Background and rationale {6a}

Inadequate and less diverse diets during critical life stages, including pregnancy, lactation, and infancy, are major contributors to childhood malnutrition, particularly in low- and middle-income countries (LMICs) [29]. The first thousand days post-conception are recognized as a critical period of potential intervention to improve early childhood development and reduce stunting [9, 28, 45]. Maternal nutritional status has been shown to directly affect the occurrence of child stunting, as well as indirectly affect stunting through other factors [44]. Research has revealed that intrauterine growth faltering is a more significant problem than previously realized [11] with approximately 20% of stunting occurring in utero in LMICs [6, 41]. Empirical studies consistently show that fetal growth restriction often originates in utero and is intrinsically linked to maternal nutrition [13, 32]. In addition to its role in child development, maternal nutrition is a key determinant of overall maternal and neonatal health, influencing pregnancy outcomes, fetal development, and birth outcomes [27]. This underscores the pivotal role of maternal diet in improving both maternal and fetal health outcomes.

The increase in nutritional demands during pregnancy and lactation is well established. Pregnant women have higher requirements for certain micronutrients such as iron, magnesium, and zinc [39]. Evidence indicates that poor maternal nutrition is responsible for around 800,000 neonatal deaths each year [4, 34]. Maternal anemia and calcium deficiency are key factors linking maternal nutrition to maternal mortality and adverse pregnancy outcomes. In LMICs, maternal anemia is estimated to contribute up to 12% to low birth weight cases, 19% to preterm birth cases, and 18% to perinatal mortality [42].

Consequent to these, there observations have been significant emphasis on improving nutrition during the critical first 1000 days from conception to 2 years of life. However, many of these efforts have focused on the fortification of foods, including US Title II food aid products. Blended flours, such as corn soy blends (CSB), have been a staple of development efforts and food aid programs, and the increased understanding of the critical role of animal proteins and micronutrients has led to the fortification of these with essential vitamins and minerals and milk powder, often referred to as CSB+ and CSB++ or super cereal, respectively [50]. These fortified products are used widely to try to address the nutritional needs of both women and children [24].

In LMICs, typical diets are less diverse and often lack animal source foods (ASF), which are a rich source of bioavailable macro and micronutrients. This deficit is often because of barriers specific to LMICs (e.g., cost, poor supply chains, food insecurity, cultural norms) [1, 31]. Current food formulations (e.g., CSB+ , supercereals) are designed to enhance nutritional density and bioavailability. Notably, these nutritional attributes are naturally present in most ASF. The nutritional value of ASF has been highlighted as an important dietary tool in efforts to reduce child malnutrition due to high nutrient density and bioavailability of macronutrients [36], as well as essential micronutrients (vitamin B12, iron, choline, zinc, and iodine), which may be suboptimal in vegetarian diets [31]. Adequate amino acid supply is also important, especially during growth and development. In a meta-analysis by Pimpin et al. [40], both maternal and infant supplementation of animal protein was found to increase birthweight and young children’s weight. Given these nutritional advantages of ASF when compared to plant-based diets, there is an increasing dialogue around efforts to increase ASF in the diet, particularly in smallholder farming communities in LMICs [37], where dietary diversity is typically low and nutritional outcomes are poor [1, 10].

Among ASF, eggs are an excellent source of protein, essential fatty acids, and vitamins A and B12, as well as choline and docosahexaenoic acid (DHA), nutrients associated with child growth and cognitive development [21, 26]. Like other ASF across LMICs, eggs are rarely consumed among populations of women of reproductive age in sub-Saharan Africa, particularly in rural areas where rates of malnutrition are high, despite the relative affordability and availability of eggs compared to other ASF [1, 26].

Studies that examined eggs as a dietary tool to reduce malnutrition have focused on child egg consumption (at 6 months of age or later) or as part of a comprehensive package combined with other supplements [48] with little research exploring exclusively the impact of eggs among pregnant women as a mechanism to improve both maternal and child health outcomes. Consumption of eggs by infants and young children as a complementary food has been found to improve nutritional outcomes [22, 25, 30, 35] and early child development [14] in LMICs. Importantly, however, other studies have found that initial positive results in child growth outcomes following egg consumption were not sustained [20], and others still have found no impact [43, 47]. Another study by Pasqualino et al. [38] found no significant effect of egg supplementation in children (6–12 months) on length for age (LAZ) but did find a significant effect on weight.

Given the alignment of nutrient demand during pregnancy and the composition and bioavailability of nutrients in ASF, consumption of ASF during pregnancy has the potential to improve fetal growth in regions where malnutrition is prevalent. Further, because eggs prepared as hard boiled are safe and do not increase the risk of exposure to pathogens [15], and their nutrient profile indicates a potential to improve brain development, consumption of eggs during pregnancy has little to no risk [49] and offers other potential benefits to maternal and child development. Against this background and considering the existing lack of evidence, this randomized control trial (RCT) will address an existing gap in the literature surrounding the potential effect of maternal consumption of eggs on in-utero growth and brain development.

## Hypotheses and objectives {7}

The study hypothesizes that women who consume two eggs a day will have children whose LAZ at birth is significantly higher than LAZ among those born to women who consume a typical diet. The typical diet in Rwanda often includes fortified blended flour (FBF), a mixture of corn and soy with added vitamins and minerals, which is commonly provided by the government through various nutrition programs for children, undernourished pregnant women, mothers, and other vulnerable groups. FBF is often shared within the family. Therefore, all pregnant women in the study will receive CSB++ (if not already receiving from the government), and the comparison of the effects of eggs will be over a diet that consumes CSB+. The main objective of the study is to determine whether maternal egg consumption during pregnancy improves a child’s growth, which will be measured at birth, 6 weeks, and 6 months of age. The trial also aims to answer additional research questions related to maternal and child health, including the following:

*Maternal outcomes*

- Does maternal egg consumption lead to improved maternal health indicators during pregnancy (including weight gain, blood pressure, anemia, and body mass index (BMI)) when compared to a typical diet?
- Do women who consume eggs during pregnancy experience reduced levels of anemia following birth compared to those who consume a typical diet?
- Does maternal egg consumption lead to improved prenatal and postpartum mental health of mothers, when compared to typical diet?

*Child outcomes*

- Does maternal egg consumption lead to improved birth outcomes when compared to birth outcomes of mothers who consume a typical diet (birth outcomes will include miscarriage, stillbirth, preterm births, low-birth weight, small-for-gestational age)?
- Does any observed effect of maternal egg consumption on birth outcomes (compared to typical diet) differ by child sex (male/female)?
- Does maternal egg consumption improve other child growth outcomes (including stunting, wasting, and underweight) at 6 months of age compared to a typical diet?
- Does maternal egg consumption lead to improved levels of micronutrients (iron, vitamin B12, vitamin A) in children at 6 months of age compared to a typical diet?
- Does maternal egg consumption lead to improved neurodevelopment in utero (as indicated by ultrasound-based measures) or during the first 6 months of life (head circumference, Hammersmith Neonatal Neurological Exam (HNNE), and Caregivers-Reported Early Development Instrument (CREDI) scores compared to typical diet?

## Trial design {8}

**Fig. 1:**
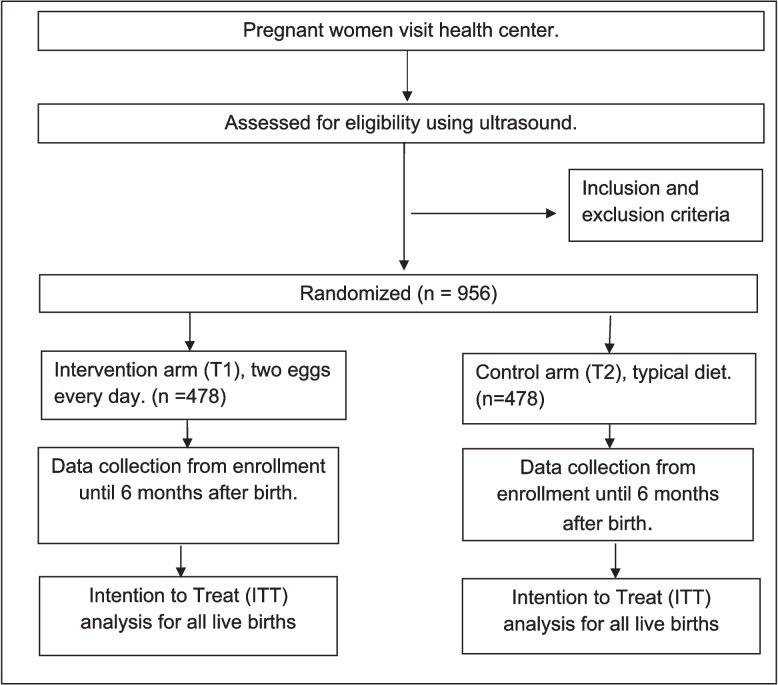
Study design of the *Deux Oeufs* randomized controlled trial

The study is a randomized controlled trial (RCT) with parallel 1:1 assignment of participants to two study arms: (T1) two eggs per day (intervention) or (T2) typical diet (control). Block randomization will be stratified by gestational age group (09 < 11 weeks and 12 < 14 weeks). Women in the intervention arm will be asked to consume two eggs per day under the direct observation of study staff at a community health worker (CHW) home. Participants in the control arm will be asked to consume their typical diet, including CSB+. All participants will continue to receive standard care for pregnant women (antenatal care and institutionalized births among others), as outlined by the GoR. In addition, the study will provide a mobile phone, family health insurance (if not already covered), and modest compensation for participation. The amount of compensation was determined based on formative research and approved by the Rwandan National Ethics Committee (RNEC). The compensation for each engagement at the clinic (first, second, and third trimester visits, birth and two postpartum visits, at 6 weeks and 6 months of child age) was decided to be 3000 RWF. They will also be provided with transportation reimbursement for the study visits that occur at the health centers. All participants not receiving Shisha Kibondo (government brand for CSB++) from the GoR as part of their standard prenatal care will receive the same amount (6 kg/month) from the study. Figure 1 illustrates the study recruitment flow and treatment allocation.

## Methods: participants, interventions, and outcomes

### Study setting {9}

The study will take place in *Nyagatare* District in the Eastern Province of Rwanda. *Nyagatare* District was selected because it (1) has a relatively high rate of stunting among children under 5 (30%) [33], (2) is within the USAID Feed the Future Zone of Influence, and (3) is not a district or region with the highest rates of malnutrition, where extensive and evolving government and nongovernmental nutritional interventions would challenge operational activities and the interpretability of the study’s findings. Pregnant women living in the catchment areas of *Ndama* Health Center in *Karangazi* sector will be the targeted population. *Ndama* Health Center was purposively selected as the study site because it serves a primarily rural population, and the reported historical number of births within the catchment area will provide a sufficient number of births for the study to meet enrollment targets. The selection of the study site was based on formative field work conducted in collaboration with the University of Rwanda and World Vision Rwanda from July through September 2023 and in concert with local, district, and national health officials.

### Eligibility criteria {10}

All pregnant women will be considered for enrollment, as per the eligibility criteria below.

#### Inclusion criteria

- Lives in the study area (catchment area of *Ndama* Health Center)
- Ultrasound confirmed viable intrauterine pregnancy
- Aged between 18 and 44 years
- Ultrasound confirmed gestational age of 9 up to 13 weeks + 6 days
- Agrees to participate and provides informed consent

#### Exclusion criteria

- Intention to move outside the study area in the next 12 months
- Intention to deliver at a location outside of the Eastern province
- Any known adverse reaction to eating eggs or porridge ingredients
- Any condition that could affect the ability to comply with the study requirements or understand the informed consent process
- Participation in other clinical trials (to avoid the burden and potential interactions between interventions)
- Inability or unwillingness to comply with the study requirements, such as attending regular follow-up visits or adhering to prescribed nutritional supplementation

### Who will take informed consent? {26a}

The Rwanda National Ethics Committee (RNEC) and the Institutional Review Board (IRB) of the University of Florida approved the *Deux Oeufs* study. The study will fully comply with the code of ethics regarding participant enrollment, study assessment, and data protection. Trained World Vision study staff will administer an initial informed consent for screening, before performing a verbal eligibility assessment and conducting an ultrasound screening. When a study subject is screened for enrollment and is deemed eligible, she will be offered enrollment and engaged in a full informed consent process.

During the informed consent process, a study team member will communicate the study’s purpose, the assessments involved, the study process, and the potential benefits and risks involved to the participants in nontechnical language. Participants will have ample time to consider the study’s implications before deciding whether to enroll. Those who choose to participate must sign and date an informed consent form. In case of illiteracy, the study staff will ask for a thumbprint. Enrolled participants may withdraw from the study at any point in time. The original and signed informed consent forms will be retained with the participants’ records, and the participant will be provided with a copy of the signed consent form, as well as an information sheet, per RNEC requirements.

If any changes or amendments to the approved protocol directly affect a participant’s decision to continue in the study, the informed consent form will be amended accordingly. Participants will be required to re-sign the revised informed consent form after the same has been approved by the ethical committees of RNEC and the IRB at the University of Florida.

### Additional consent provision for collection and use of participant data and biological specimens {26b}

On the informed consent form, the research team will also seek participants’ consent to provide hair and blood samples, and for the study team to share the relevant data with regulatory authorities and IRB, where appropriate. Hair samples will be collected from mothers to assess cortisol levels, a hormone indicative of stress.

## Interventions

### Explanation for the choice of comparators {6b}

The intervention group will receive a daily supplement of two eggs (with consumption directly observed) from enrollment in the first trimester through childbirth. Both the intervention and control groups will receive standard information, education, and communication (IEC) about healthy diets and appropriate care during pregnancy. Participants will not be restricted from adding eggs or any other food to their diet.

All enrolled participants will receive the Government of Rwanda’s (GoR) up-to-date standard care for pregnant women, a mobile phone, health insurance (if not already covered), and modest compensation for traveling to a community health worker’s (CHW) home daily for study engagement. Additionally, any pregnant woman not already receiving the government-provided fortified corn-soy blend (CSB++ or *Shisha Kibondo*) will receive an equivalent amount (6 kg) from the study.

In order to uphold ethical considerations and account for potential spillover effects of the intervention, the study design does not include a true control group. The control group will receive the same level of care, information, education, and communication (IEC), and equal amount of CSB+; only egg supplementation will be different between the two groups. This design allows for a clear comparison of the effects of egg supplementation on pregnancy and child growth outcomes, beyond the benefits of a typical diet that includes CSB+ .

### Intervention description {11a}

All women enrolled in the study will be asked to visit a designated village-based consumption site (CHW’s home) daily. Sites must be within a 20-min walk from women’s homes. When a CHW house is not within a 20-min walk, additional sites (such as the home of a World Vision volunteer) will be identified close to the participant’s house. Study staff will prepare and transport hard-boiled eggs daily to CHWs’ houses (or designated consumption site), where CHWs will distribute eggs to the women in the intervention group every day. Study staff will observe and record consumption (no consumption, partial, or complete). The study staff will also ask about 24-h recall of quantity and consistency of CSB+ consumption and any adverse reaction or allergy to eating eggs. Women in the treatment arm and the control arm will both receive IEC communication. All women (treatment and control) will be compensated for their daily travel to the CHW’s house. The daily compensation of 500 RWF per day will be paid on a weekly basis if women attend at least 6 days in a week. However, in cases of excused absence, they will receive daily compensation for those days.

Participants in both the intervention and control groups will receive guidance to maintain their usual diets outside of the study intervention. No dietary restrictions will be imposed.

### Criteria for discontinuing or modifying allocated interventions {11b}

The intervention will be discontinued in cases of formal withdrawal, loss of follow-up, severe illness preventing study participation, and adverse events. If there is a loss of pregnancy, the woman in the egg group will continue to receive eggs for 30 days after miscarriage or fetal death if she wishes to. However, no daily cash compensation will be provided after the loss of pregnancy. Otherwise, the intervention (egg consumption) will end at childbirth.

### Strategies to improve adherence to interventions {11c}

Adherence to the intervention will be monitored throughout the study to ensure high uptake, with the goal of 100% adherence (daily observed consumption of 2 eggs per day, 7 days a week). Although 100% adherence is desired, the study team recognizes that the woman may not be present on certain days due to travel outside of the study area, work or family obligations, sickness, or other reasons. The woman also has the option of refusing the food on any given day. The study team will seek to document planned absences to anticipate the resumption of the nutritional supplement upon the woman’s return. To improve adherence, the women will also receive a small compensation for their daily visits to CHW houses (500 RWF per day) if they come at least 6 out of 7 days. However, in case of planned and excused absence, women will receive daily compensation for those days. The daily compensation amount was pilot tested in January and February 2024 to ensure that the payment was both motivational and effective. The compensation amount was then approved by ethical authorities (both Rwanda and UF) to be non-coercive. When a participant fails to present to the study team to receive her assigned supplement without prior notification of absence or explanation, a team member will attempt a visit to the village or study household later in the day to ensure adherence. Cell phones are provided by the study to optimize communication between the study team and all participating women. The food delivery staff who provide the eggs will attempt to reach women by telephone if they do not attend as expected, encouraging them to come to the CHW home, even later than designated. At the end of the day, however, if a participant has not come to the CHW home to receive the eggs, the staff may travel to her home to try to deliver and observe egg consumption. This will occur any day that the woman does not attend at the CHW home for egg consumption during the designated window but is considered a secondary option given the complexity around food sharing that may occur with the provision of eggs directly at homes.

If a participant is hospitalized and remains within the district of *Nyagatare*, the study team will deliver eggs to the facility, with the permission of health facility staff. Additionally, if the participant misses food supplement due to absence, refusal, or another reason but has not formally withdrawn from the study, the study team will attempt to contact the mother by phone or through CHW outreach every 2–4 days for up to 1 month to encourage re-engagement. If the participant re-engages with the study team, all intervention and data collection will be reinstated. Any barriers to participation identified during the study will be considered and addressed as much as possible by the research team.

### Relevant concomitant care permitted or prohibited during the trial {11d}

Participants will not be restricted from adding eggs or any other food to their diet.

### Provisions for posttrial care {30}

Although the study intervention (egg provision) ends at childbirth, women and infants will continue to engage in the study through 6 months of age. Study staff will conduct follow-up visits at 6 weeks of age and 6-month postpartum to collect additional information on maternal and child health outcomes. The study team intends to seek funding for extended follow-up to track child development up to 2 years of age.

### Outcomes {12}

#### Primary outcome

The primary outcome that will be used to assess the impact of egg supplementation during pregnancy on child malnutrition is newborn birth length, as indicated by LAZ using INTERGROWTH 21st standards.

#### Secondary outcomes

A list of secondary outcomes of the trial can be found in Table 1.

**Table 1:** Outcomes of *Deux Oeufs* trial

	**Outcome**	**Type/metric**	**Measurement method**	**Timepoint**	**Aggregation**
***Primary outcome***	Newborn length for age z-score	Continuous (z-score)	INTERGROWTH 21st growth standards	Within 72 h of birth	Mean (SD)
***Secondary outcomes***					
*** Child outcomes***					
* In-utero measurements*	Head circumference z-scores	Continuous (z-score)	INTERGROWTH 21st growth standards	Between 20–26 weeks and 30–34 weeks of gestational ages	Mean (SD)
	Femur length z-scores	Continuous (z-score)	INTERGROWTH 21st growth standards	Between 20–26 weeks and 30–34 weeks of gestational ages	Mean (SD)
	Abdominal circumference z-scores	Continuous (z-score)	INTERGROWTH 21st growth standards	Between 20–26 weeks and 30–34 weeks of gestational ages	Mean (SD)
	Bi-parietal z-scores	Continuous (z-score)	INTERGROWTH 21st growth standards	Between 20–26 weeks and 30–34 weeks of gestational ages	Mean (SD)
	Estimated fetal weight z-scores	Continuous (z-score)	INTERGROWTH 21st growth standards	Between 20–26 weeks and 30–34 weeks of gestational ages	Mean (SD)
	Cerebellar diameter	Continuous (cm)	Ultrasound measurement	Between 20–26 weeks and 30–34 weeks of gestational ages	Mean (SD)
	Corpus callosum length	Continuous (cm)	Ultrasound measurement	Between 20–26 weeks and 30–34 weeks of gestational ages	Mean (SD)
	Thalamus midline axial	Continuous (cm)	Ultrasound measurement	Between 20–26 weeks and 30–34 weeks of gestational ages	Mean (SD)
	Gangliothalamic ovoid diameter	Continuous (cm)	Ultrasound measurement	Between 20–26 weeks and 30–34 weeks of gestational ages	Mean (SD)
	Gangliothalamic ovoid sagittal length	Continuous (cm)	Ultrasound measurement	Between 20–26 weeks and 30–34 weeks of gestational ages	Mean (SD)
	Gangliothalamic ovoid estimated volume	Continuous (cubic cm)	Ultrasound measurement	Between 20–26 weeks and 30–34 weeks of gestational ages	Mean (SD)
* Newborn measurements*	Small for gestational age (SGA) at birth (< 10th percentile and < 3rd percentile)	Binary (two thresholds)	INTERGROWTH 21st growth standards	Within 72 h of birth	Proportion
	Birthweight for gestational age at birth	Continuous (z-score)	INTERGROWTH 21st growth standards	Within 72 h of birth	Mean (SD)
	Preterm live birth (< 37 weeks completed gestation)	Binary	Delivery < 37 + 0 weeks completed gestation	At birth of newborn	Proportion
	Birthweight (g)	Continuous (in g)	Measured using calibrated scale	Within 72 h of birth	Mean (SD)
	Low birthweight	Binary	Birthweight < 2500 g	Within 72 h of birth	Proportion
	Birth weight-for-age z-score	Continuous (z-score)	INTERGROWTH 21st growth standards	Within 72 h of birth	Mean (SD)
	Small vulnerable newborn status	Binary	Any of: SGA (< 10th), preterm (< 37 weeks), or low birthweight (< 2500 g)	Within 72 h of birth	Proportion
	Head circumference z-score	Continuous (z-score)	INTERGROWTH 21st growth standards	Within 72 h of birth	Mean (SD)
* Infant measurements*	Prevalence of stunting among infants	Binary	Length-for-age < −2 SD according to the WHO Multicenter Growth Reference Study standards	At 6 weeks and 6 months of age	Percentage
	Infant weight-for-age z score	Continuous (z-score)	WHO Child Growth Standards	At 6 weeks and 6 months of age	Mean (SD)
	Prevalence of underweight	Binary	Weight-for-age < -2 SD according to the WHO Multicenter Growth Reference Study standards	At 6 weeks and 6 months of age	Percentage
	Child neurodevelopment- HNNE	Continuous/domain score	Hammersmith Neonatal Neurological Examination (HNNE)	At 6 weeks of age	Domain and total scores
	Child neurodevelopment-CREDI	Continuous	Caregiver-Reported Early Childhood Development Instrument (CREDI) score (long form)	At 6 months of age	Domain-specific scores
*** Maternal outcomes***					
	Maternal weight	Continuous (in kg)	Measured using calibrated scale	At enrollment (between 9–< 14-weeks gestation), 20–26-weeks gestation, 30–34-weeks gestation, and during labor/delivery	Mean (SD)
	Maternal weight gain	Categorical (inadequate/adequate/excessive)	IOM guidelines and calculated based on maternal weight at labor/delivery-maternal weight at enrollment	At enrollment (between 9–< 14 weeks gestation), 20–26-weeks gestation, 30–34-weeks gestation, and during labor/delivery	Proportions
	Maternal prenatal and postnatal depression	Continuous and binary	Edinburgh Postnatal Depression Scale (EPDS) (range 0–30; 12 or higher defining maternal depression)	At 6 weeks and 6 months of child age	Mean (SD), prevalence above threshold
	Maternal cortisol concentration	Continuous	Cortisol concentration from maternal hair samples	Between 9–< 14 weeks, 20–26 weeks, and 30–34 weeks gestational ages, at time of birth, and at 6 weeks and 6-months post birth	Mean (SD)
	Minimum Dietary Diversity for Women (MDD-W)	Binary	Diet Quality Questionnaire (DQQ) will be used to generate MDD-W, used here as meeting the validated threshold or not 0–10 scale, where a higher score indicates more diversity (better) with threshold of having at least 5 to have met minimum requirements (coded as 0 for not having met/1 for having met MDD)	Between 9–< 14 weeks, 20–26 weeks, and 30–34 weeks of gestational ages, at time of birth, and at 6-week and 6-month post-birth	Proportion
	Dietary diversity score	Continuous	Sum of score from different food groups 0–10	Between 9– < 14 weeks, 20–26 weeks, and 30–34 weeks of gestational ages, at time of birth, and at 6-week and 6-month post-birth	Mean DDS
	Maternal hemoglobin	Continuous/categorical	Trimester-specific WHO cutoffs	Between 9– < 14 weeks, 20–26 weeks, and 30–34 weeks of gestational ages, at time of birth, and at 6-week and 6-month post-birth	Mean (SD), anemia prevalence by trimester
	Biomarkers of micronutrient status, growth factors, systemic inflammation, environmental enteric dysfunction, metabolomics, choline, and lipid panel (including HDL and LDL cholesterol)	Continuous	Measured using whole blood or serum samples among women and infants	Between 9– < 14 weeks, 20–26 weeks, and 30–34 weeks of gestational ages, at time of birth, and at 6-week and 6-month post-birth	Mean (SD)
	Maternal blood pressure	Continuous/categorical	Diastolic and systolic blood pressure	Between 9–< 14 weeks, 20–26 weeks, and 30–34 weeks of gestational ages, at time of birth, and at 6-week and 6-month post-birth	Mean (SD), prevalence of hypertension per protocol
*** Other outcomes***					
	Thalamus length, width, and transverse	Continuous (in cm)	Ultrasound measurement	Between 20–26 weeks and 30–34-week gestational ages	Mean (SD)
	Consumption of ASF	Score (0–4)	Based on DQQ questionnaire	Between 9– < 14 weeks, 20–26 weeks, and 30–34 weeks of gestational ages, at time of birth, and at 6-week and 6-month post-birth	Mean (SD)
	Child’s motor, cognitive, socio-emotional, and language development domain specific scores	Domain-specific scores	Based on CREDI long form	6 months of age	Mean (SD)

### Participant timeline {13}

Women will be recruited during the first trimester of pregnancy and will be asked to plan to remain in the study until the child is 6 months of age. Women will engage in study visits at the health center during the first (enrollment), second, and third trimesters of pregnancy. Additional follow-ups will be conducted at the time of birth (within 72 h) and when the child is 6 weeks and 6 months of age at the health facility. In addition, after enrollment, all participants will be asked to come to the CHW house every day to receive IEC messaging and eggs (intervention group only). Table 2 details participants’ timeline and assessments for the study.

**Table 2:** Timeline of enrollment, intervention, and data collection from *Deux Oeufs* participants

	**During pregnancy**			**Birth**	**Post-birth follow-up**	
**Timepoint**	**GA (9– < 14 weeks)**	**GA (20–26 weeks)**	**GA (30–34 weeks)**	**Within 72 h**	**6 weeks**	**6 months**
**Enrollment**						
Eligibility screen	X					
Informed consent	X					
**Intervention**						
Two eggs supplement (treatment group)	X	X	X	X		
Daily health messages (treatment group and control group)	X	X	X	X		
**Assessments**						
Ultrasound assessment	X	X	X			
Blood sample collection (women) and assessment (RBC, Hemoglobin, cholesterol, MCV, and platelets)	X	X	X		X	X
Blood sample collection (infant)					X	X
Hair sample collection (mother)	X	X	X		X	X
**Baseline and follow-up surveys**						
Mother’s anthropometry	X	X	X	X*	X	X
Mother’s clinical assessment (blood pressure and current health condition)	X	X	X			
Mother’s medical and obstetrics history	X					
Socioeconomic status	X					X
Smoking and alcohol consumption	X	X	X		X	X
Dietary Quality Questionnaire (DQQ)	X	X	X		X	X
Household Food Security (HFIAS)	X					X
Water and sanitation	X					
Pregnancy history	X					
Malaria prevention	X					
Physical activity	X	X	X			
Depression (EPDS)		X	X		X	X
Intention to breastfeed		X	X			
Immediate feeding				X		
Gender-based violence					X	X
**Outcomes**						
Child anthropometry				X	X	X
Child health status and medical history after birth			X		X	X
HNNE					X	
Child development (CREDI)						X
Infant feeding practices				X	X	X
Maternal biomarkers	X	X	X			
Child biomarkers					X	X
*< 1 week before birth						

### Sample size {14}

Nutritional interventions during pregnancy have yielded mixed results in their impact on LAZ at birth. Previously reported mean difference in LAZ ranges from 0.12 [2, 18] to 0.29 [12, 17]. Notably, the 0.29 effect size observed by Dhaded et al. [12] was associated with nutritional supplementation initiated prior to conception, whereas supplementation beginning in the first trimester yielded a smaller effect size of 0.21. Considering most studies report an effect size below 0.20 and based on the available literature on other food supplementation as found in some literature review papers by Imdad and Bhutta [23] and Stevens et al. [46], we hypothesize a mean difference of 0.18 in LAZ to be both a reasonable and clinically meaningful effect size. As such, we calculate the sample size based on testing two hypotheses simultaneously:

$$H0:d1\leq0\;vs.\;Ha:d1>0$$

For our hypothesized effect size, the sample size required with 20% attrition (accounting for pregnancy loss, stillbirths, and loss of follow-up) to attain an 80% marginal power with a 5% level of significance was 478 per arm, based on a two sample one-sided test. Thus, we aim to enroll an overall sample of 956 women in the proposed study. Sample calculation was performed using “pwr” package [5] in RStudio.

### Recruitment {15}

Before enrollment begins and during the entire study period, study staff will organize educational and informational campaigns in villages within the catchment area to promote early antenatal care (ANC) visits and facilitate visits to health centers. This is necessary in order to identify eligible women within the first trimester of pregnancy. CHWs and other identified community champions will encourage women in the village who are of childbearing age to attend ANC visits as soon as they suspect or confirm they are pregnant. Formative research indicated that many women delay health-seeking behavior around pregnancy until they have a high level of confidence that they are pregnant. Study staff, together with CHWs, will also raise awareness of the symptoms of early pregnancy and about the study in general through sector social affairs officers, cell-level officials, and village chiefs and by posting informational posters at health centers, health posts, and other appropriate locations. Urine-based pregnancy tests will be distributed to CHWs to encourage discrete/private confirmation of suspected pregnancy and attendance at a first antenatal care visit as early as possible. Study personnel will work with CHWs who keep a roster of all pregnancies in the village. CHWs will identify potential participants in their first trimester and encourage them to visit the health center for the study. At the commencement of the study, staff will coordinate with CHWs to compensate transportation costs for women with positive pregnancy tests from the village to the health care center serving the catchment area. The study staff will be available at the health center to conduct standardized screening ultrasounds and gather baseline data. Women who present at the health center for an ANC visit without referral from a CHW may also engage in the screening and enrollment process previously described.

For ultrasound screening, a portable handheld ultrasound system that combines GE VScan Air with the IntelligentUltrasound artificial intelligence-enabled ScanNav FetalCheck (Cardiff, Wales) will be used to date pregnancies. Pregnancy will be dated using ScanNav FetalCheck. If a FetalCheck is unable to provide a measure (typically < 10-week gestation), crown-rump length (CRL) will be used to date pregnancy in conjunction with the reported last menstrual period based on standard methods and INTERGROWTH 21st standards. Once eligibility is confirmed, participants provide informed consent before enrollment and assessment during an initial study visit.

Participants will be asked to visit their health center according to a normal ANC visit schedule. At two of these ANC visits, the participants will be examined and surveyed by our study team: one at 20–26 weeks and another at 30–34 weeks. The study staff will provide an approximate appointment date for these visits, and participants will be reminded of the same by phone in the days leading up to their second and third trimester visits.

## Assignment of interventions: allocation

Following enrollment, women will be randomly allocated to the control arm or the intervention arm by selecting an envelope of their choice from three options.

### Sequence generation {16a}

The study will apply stratified block randomization with varying block sizes to randomly assign the treatment arms and to preserve the balance of treatment across gestational age groups (9–11 weeks and 12 < 14 weeks). A chain of blocks will be pre-generated for each stratum, e.g., 1000 blocks. The size of each block will also be randomly and independently chosen as 2, 4, or 6 individuals. The study will use the tool at sealedenvelope.com to generate the randomization list. The random seed will be generated using the random integer generator at random.org, which introduces randomness from unpredictable atmospheric noise (see more below).

### Concealment mechanism {16b}

Each assignment will be printed and stored in a sealed envelope at the University of Florida, and envelopes will be ordered according to the randomization list in each gestational age group. When a study subject is screened for enrollment and is deemed eligible, she will be offered enrollment and engaged in an informed consent process. If she agrees to participate (and provides consent), she will choose an envelope from among three arranged consecutively, each of which has a piece of paper in it that indicates whether she is in the treatment or control group. The choice of envelope was included with the block design to ensure a sense of autonomy among participants and that they feel able to make a choice. Each subject will have a unique randomization code, in addition to a unique study ID, so that the original assignment of each subject can be easily tracked.

### Implementation {16c}

A biostatistician on the study team (Y. Y.) will generate the allocation sequence and be blinded to the assignment. Study staff who take the informed consent will enroll participants. Separate study staff will be responsible for facilitating each participant’s selection of an envelope and allocation into control or intervention groups.

## Assignment of interventions: blinding

### Who will be blinded? {17a}

Field staff responsible for measuring primary and secondary outcomes and data analysts performing the analysis will be blinded about treatment assignments. Study staff involved in administering informed consent and data collection (except daily compliance) will also be blinded. The assignment envelopes will be pre-filled in advance, and both the study participant and the staff person facilitating this randomization reveal process will be blinded to what is inside the envelope until it is opened. The two primary study analysts responsible for the analysis of the primary outcome and replication will also be blinded until replication has been confirmed.

### Procedure of unblinding if needed? {17b}

Staff conducting data quality checks and staff doing the intervention delivery will only be unblinded to the allocated intervention. All other study staff will remain blinded.

## Data collection and management

### Plans for assessment and collection of outcomes {18a}

At enrollment, an ultrasound screening will be conducted to determine eligibility, followed by a baseline survey on demographic and background characteristics of the household. Questions in the survey will include pregnancy and medical history, maternal behavior towards risk factors (e.g., malaria), physical activity, dietary diversity using the Dietary Quality Questionnaire (DQQ) (Global Diet Quality Project), household food security using the Household Food Insecurity Access Scale (HFIAS) [8], and water and sanitation practices. Women’s anthropometric measurements will be taken using Seca scales, including height (Seca 213 portable measuring rod), weight (Seca 874), and mid-upper arm circumference (MUAC). Blood samples and hair samples will also be collected at baseline.

During two additional clinical visits in the second (20–26 weeks) and third trimester (30–34 weeks), anthropometric measurements (height, weight, MUAC) from all women will be taken, as well as blood and hair samples. Ultrasound measurements will also be taken to assess fetal growth measures that include biparietal diameter, head circumference, abdominal circumference, and femur length. Information on medical conditions since the last visit, depression screening using the Edinburgh Postnatal Depression Scale (EPDS), physical activity, breastfeeding intention, consumption and preparation recall of CSB+, and maternal diet (DQQ) will be collected using a short survey during these visits.

At birth, anthropometric measurements of all neonates will be assessed twice within 72 h after birth, with the first 24 h as the target window. A third measurement will be taken in case of discrepancies between the first two measurements. For recumbent length, a third measurement will be taken if the difference between the first two measurements is more than 0.7 cm. A third weight measurement will be taken in case the difference in the weight in the first two measurements is more than 10 g. Length measurements will be taken using length board S0114540 set3, and weight will be measured using Seca 334 scale.

After birth, the mother and child will be asked to engage with study staff for additional assessments during the child’s routine health exams at 6 weeks and 6 months of age. At each of these visits, the mother will answer survey questions including the DQQ adapted for Rwanda [19], infant feeding practices, and whether the infant is exclusively breastfed [52]. The questionnaire will use WHO-recommended core indicators [52] of optimal breastfeeding that include early initiation of breastfeeding (within 1 h of birth) and exclusive breastfeeding until 6 months after birth. A survey module on the infant’s health and morbidity (caregiver-reported diarrhea, respiratory issues, fever, malaria) and care-seeking behavior will also be administered. If consented and pending funding availability, medical records at health facilities (outpatient, integrated management of childhood illness (IMCI), inpatient) and CHW sick child encounter forms will be extracted to understand the impacts of the nutrition interventions on child health outcomes, including diarrhea, respiratory infections, fever, and malaria, as well as mortality. The study team will also administer survey questions regarding exposure to gender-based violence (using questions from the Demographic Health Surveys).

At birth, 6 weeks, and 6 months of age, the child’s recumbent length will be measured twice using length boards to the nearest 0.1 cm. Weight will be measured to the nearest 0.1 kg using a digital scale (Seca 334). A third measurement will be taken in case of discrepancies between the first two measurements. For length, a third measurement will be taken if the difference between the first two measurements is more than 0.7 cm. A third weight measurement will be taken in case the difference in weight between the first two measurements is more than 10 g. The two most approximate readings will be averaged for analysis. Head circumference of the infant will be measured to the nearest 0.1 cm using a standard, non-stretchable measuring tape, ensuring accurate and consistent placement around the largest part of the infant’s head (above the eyebrows and ears and around the back of the head) for reliability across measurements. MUAC will also be assessed at 6 months of age using a calibrated, non-stretchable measuring tape placed around the mid-upper arm at the midpoint between the shoulder and elbow, with measurements recorded to the nearest 0.1 cm to monitor nutritional status, and referrals will be made for those classified as moderate or severe acute malnutrition.

Additionally, at 6 weeks of age, infants will be assessed for neurodevelopment using the Hammersmith Neonatal Neurological Examination (HNNE) for newborn development assessment and at 6 months using the Caregiver Reported Early Development Instruments (CREDI), a validated assessment tool [51] used previously in Rwanda [7]. The CREDI (long form) provides domain-specific scores in motor, cognitive, language, and social-emotional development, as well as an overall score. Blood will be drawn from both the mother and the infant at 6 weeks and 6 months and stored as described below. A hair sample from the mother will be requested if consented.

### Plans to promote participant retention and complete follow-up {18b}

Study follow-up visits will coincide with participants’ routine visits to the health clinics. Pregnant women in *Nyagatare* District are encouraged to visit health centers every month during their pregnancy. The *Deux Oeufs* study will conduct visits for the trial during two of these scheduled visits at approximately 20–26 weeks and 30–34 weeks of gestation. Participants will also receive a text and phone reminder prior to each visit. The women also visit health clinics at approximately 6 weeks and 6 months after birth for infant vaccination and a well-child exam. The study team will schedule study visits with these routine appointments. Transportation to and from these visits is provided by the study, thus increasing not only retention in the study but also routine ANC and well-child health visits.

### Data management {19}

All data, except biological samples and ultrasound images, will be collected on encrypted tablets using the Research Electronic Data Capture (REDCap). Daily compliance data of the intervention will be collected using encrypted smartphones using REDCap. GPS location of participants’ houses without identifiable data will be collected using a handheld GPS device (Garmin 65). Regular monitoring will ensure data quality. Range checks for the data values will be automated to identify human errors while filling out the forms. All data will be transferred to a central server on a weekly basis and will be processed and analyzed with R and Stata.

### Confidentiality {27}

All data will be collected on encrypted tablets. Data marked as identifiable will be restricted to the principal investigators (PIs) and only those staff requiring access for logistics and quality control purposes. Access to the de-identified data will be granted to a statistical team for interim statistical analyses through a secure institutional portal. De-identified ultrasound images may be shared with an evaluator for quality control. The evaluator will not have access to identifiable information.

Biological samples and ultrasound images will only be marked with the participant identification number and will not have identifiable data attached to them.

Data will be de-identified and stored for future use after the completion of the study. The de-identified data set will not include any information that could lead to the identification of study participants, following the guidelines established by the HIPAA. Identifiable data will be destroyed after completion of the study. Long-term preservation of the de-identified data will ensure accessibility beyond the project’s life. After the completion of the project, the de-identified data will be deposited into the Data Repository of the University of Florida and the USAID data repository, where it can be publicly accessed. The dataset in the USAID repository will be findable and identifiable through a study digital object identifier (DOI).

### Plans for collection, laboratory evaluation, and storage of biological specimens for genetic or molecular analysis in this trial/future use {33}

As described above, maternal blood samples will be drawn at enrollment, second trimester, third trimester, and 6-week and 6-month visits. A total of 12-ml blood will be drawn from women and will be stored in three tubes (4 ml each). Maternal hemoglobin concentrations will be measured at the point of care using a Sysmex poch-100i automated hematology analyzer. Infant blood samples will be drawn at 6 weeks and 6 months of age. Drawn blood from the mother and infant will be kept in a cooler box with ice packs and, after isolating the serum, will be put into a −20 °C cooler within 8 h of collection. Within 7 days of collection, the sample will be transferred to Kigali for longer-term storage in −80 °C freezers at the University of Rwanda or Rwanda Biomedical Center. From the blood samples collected, a lipid (LDL and HDL cholesterol, triglycerides, HDLC4) and glucose panel will be measured among women at first, second, and third trimester visits and post-birth visits at 6 weeks and 6 months. These tests will be done at the *Nyagatare* district hospital using a *Roche Cobas c311* chemical analyzer.

The present study has approval for blood draws and long-term storage, but plans for further analyses are pending additional funding. Examples include the following:

- Maternal serum biomarkers of nutritional status and inflammation at third trimester and at 6-week and 6-month postpartum. These may include but are not limited to soluble transferrin receptor (sTfR), ferritin, retinol-binding protein (RBP), C-reactive protein (CRP), and alpha-1-acid glycoprotein (AGP).
- Child serum biomarkers of nutritional status and inflammation at birth and 6-week and 6-month postpartum. Pending funding, these may include but are not limited to sTfR, ferritin, RBP, CRP, and AGP.

Hair samples will be collected from women to assess cortisol levels, a hormone indicative of stress. Hair samples will be stored in zipped plastic bags at room temperature until transport for laboratory analysis.

## Statistical methods

### Statistical methods for primary and secondary outcomes {20a}

The primary analysis of the RCT will be based on a modified intention-to-treat (ITT) principle. This trial is designed to formally test the mean difference in the primary outcome of birth LAZ between the treatment arm and the control arm (T1–T2). All women who have a live birth and for whom childbirth length is measured within 72 h will be included in the analysis of the primary outcome. Cases for whom primary outcome data are not available (missing) will not be included in analyses. Descriptive analysis, such as frequency and percentages, will be reported for categorical variables, while means, standard deviation, and quartiles will be reported for continuous variables.

For secondary outcomes, the two-sample *t*-test will be used if the outcome is normally distributed with or without transformation, and the Wilcoxon rank-sum test will be used if the outcome is not normally distributed, where normality will be assessed using the Shapiro–Wilk test and Q-Q plot. Kruskal–Wallis and two-sample rank sum tests will be used if their distribution is non-normal. For both primary and secondary outcomes at birth, exploratory analyses will be conducted using multivariable linear or logistic regression models to adjust for potential confounders (such as baseline SES, gestational age at birth, mother’s education, mother’s age, and dietary diversity) that may be imbalanced across treatment arms. Imbalance of baseline factors may occur as a post-randomization selection bias or by chance alone. Post-randomization selection bias is possible, especially given that participants are not blinded. If this occurs, it will be crucial to adjust for imbalanced confounders in all regression analyses. Village-level heterogeneity will be considered using random effects. Spatial correlation in village-level random effects will be adjusted for if spatial clustering patterns in the outcomes are present. For longitudinal outcomes such as head circumference, linear or generalized linear mixed models will be used to account for heterogeneity in the intercept and slope of growth.

All statistical analyses will be conducted using R and Stata. The statistical significance for regression models will be set at *p* < 0.05 without further adjustment for multiple comparisons.

### Methods for additional analyses (e.g., subgroup analyses) {20b}

Subgroup analyses will be performed to assess effect modification of any treatment effect by dietary diversity, compliance with study supplement use, coexisting medical conditions, BMI, and infant gender on our study results, with proper attention to the effects of multiple comparisons on the ability to draw conclusions.

### Methods in analysis to handle protocol nonadherence and any statistical methods to handle missing data {20c}

Per-protocol analysis will be performed as a sensitivity analysis. Per-protocol is defined as completing 80% of the scheduled daily doses in the treatment arm from enrollment until birth. Additionally, we may include actual doses or dose groups instead of the assigned treatment arms in the regression models. Missing data will be treated as missing for the primary outcome.

### Plans to give access to the full protocol, participant level-data, and statistical code {31c}

The protocol of the study is publicly available on ClinicalTrials.gov identifier (NCT06405360). After the completion of the study, the de-identified data will be deposited into the Data Repository of the University of Florida and the USAID data repository, where it can be publicly accessed. The dataset in the USAID repository will be findable and identifiable through a study DOI.

## Oversight and monitoring

### Composition of the coordinating center and trial steering committee {5d}

The study is a single-site RCT in Rwanda. A trial manager at World Vision-Rwanda will be responsible for the day-to-day field activities and will be responsible for managing the field team, including a project facilitator, data management officer, nurse sonographers, food aid distributors, and egg preparation staff. To enhance collaboration and ensure project effectiveness, regular weekly team meetings will be held among the study team at the University of Florida, University of Rwanda, World Vision US, and the World Vision Rwanda field team. Protocols for data collection and sharing will be shared to ensure transparency and accessibility. A quarterly update will be communicated to partners in the GoR, including the National Child Development Agency, the Rwandan Biomedical Center, the Rwanda Agricultural Board, and other key stakeholders.

### Composition of the data monitoring committee and its role and reporting structure {21a}

The data monitoring committee members will include the PI, the PI at World Vision US, a data management consultant at the REDCap office within UF’s Clinical Translational Science Institute, a data manager with the field team, and a postdoc at UF. The committee will meet at least every 3 months. The committee will ensure the protection of the interests of study participants and ensure the integrity of the study conduct and results.

### Data monitoring: interim analysis {21b}

An interim analysis will be conducted after data are available for approximately 100 births and will be focused on secondary outcomes. No interim analysis will be conducted on the primary outcome (birth length) of the study. This analysis will primarily focus on feasibility, safety, and adherence to the intervention, rather than formal hypothesis testing. The principal investigator and study team will review these findings.

### Adverse event (AE) reporting and harms {22}

Any adverse event, including a clinically significant abnormal laboratory finding, symptom, or disease temporally associated with the study, will be monitored and reported in detail in the participant’s records. AEs will be coded following MedDRA terminology to ensure consistent classification, analysis, and reporting. In trial publications, we plan to report all serious adverse events (SAEs) as well as any AEs deemed related to the intervention. The PI will evaluate and follow up with all AEs and inform regulatory bodies as appropriate (RNEC and UF-IRB) of measures that will be executed accordingly.

### Frequency and plans for auditing trial conduct {23}

The monitoring and evaluation team from UF will have monitoring visits at least biannually. The monitoring team will review the study conduct and compliance with the study protocol, good clinical practice, standard operating procedures, and applicable regulatory requirements.

### Plans for communicating important protocol amendments to relevant parties (e.g., trial participants and ethical committees) {25}

Any amendments to the study protocol deemed necessary will be discussed between the PI and Co-PIs. No changes to the protocol will be implemented without prior review and documented approval from the RNEC and UF-IRB, except when required to eliminate an immediate risk to study participants.

### Dissemination plans {31a}

The study findings will be disseminated through peer-reviewed scientific journal publications and conference presentations and will be available on the project website. Results will be communicated to the participants, Ministry of Health of Rwanda, field staff, and other relevant national and international stakeholders. Results will be shared in accessible formats, such as community meetings led by local health workers and summary brochures provided in the local language.

## Discussion

This paper describes the protocol for *Deux Oeufs*, an individually randomized two-arm trial in *Nyagatare* District, Rwanda, in which pregnant women in the intervention group will consume two eggs per day under direct observation from the first trimester through childbirth. The *Deux Oeufs* study will provide evidence of the impact of egg supplementation during pregnancy on a child’s physical and cognitive development. Formative research was conducted in Rwanda before finalizing the design of the trial. Carrying out the formative research allowed the study team to identify strengths and potential pitfalls, as well as identify appropriate study sites and further engage partners and stakeholders. The findings from the formative research and details of how they informed the study protocol presented here are currently under preparation. The main strength of this protocol is that it will be the first RCT, to our knowledge, to study the effect of egg consumption in pregnant women on the nutritional outcomes of the child. The Rwandan government has identified stunting as one of the four national priorities and is currently aligning policies and programs to address malnutrition across the country [16]. Results from this study will inform those policies and programs, in both the agriculture and health sectors, on the potential for ASF to be leveraged during pregnancy to improve child health outcomes. By evaluating the effects of maternal egg consumption during pregnancy on child growth outcomes, this study aligns with public health priorities in many LMICs, including Rwanda, where stunting rates remain a health concern. Results from this clinical trial can help shape evidence-based guidelines for prenatal nutrition, potentially influencing government and NGO-led nutrition programs to incorporate affordable, locally available animal-source foods as a strategy to improve maternal and child health outcomes. However, this RCT is limited to a single study site in *Nyagatare* District, Rwanda. Stunting is multi-causal, driven by food intake, gut health, the presence of disease, hygiene, and sanitation, among other determinants [3]. The results of the trial in *Nyagatare* District, Rwanda, may differ from a similar study conducted elsewhere, where stunting may be driven by a different set of determinants. Further studies in different contexts are thus needed, no matter the outcome of this study.

## Trial status

The study protocol (version 1.0) was approved by the Rwanda National Ethics Committee (RNEC) on December 27, 2023, and soon after by the IRB of the University of Florida. The current version of the protocol presented here is version 2.0, which was approved on October 24, 2024. Participant recruitment began on May 02, 2024, with expected completion in September 2025. However, new enrollment is currently on pause due to the termination of USAID funding. The team is actively looking for additional funding, and enrollment will resume as soon as additional funding is secured.

## Data Availability

After the completion of the project, the de-identified data will be deposited into the data repository of the University of Florida and the USAID data repository, where it can be publicly accessed. The dataset in the USAID repository will be findable and identifiable through a study DOI.

## Abbreviations

ANC: Antenatal care
ASF: Animal source foods
BMI: Body mass index
CHW: Community health worker
CREDI: Caregiver-Reported Early Development Instruments
CSB: Corn soy blend
DOI: Digital object identifier
DQQ: Dietary Quality Questionnaire
EPDS: Edinburgh Postnatal Depression Scale
GoR: Government of Rwanda
HNNE: Hammersmith Neonatal Neurological Examination
IEC: Information, education, and communication
IMCI: Integrated management of childhood illness
IOM: Institute of Medicine
IRB: Institutional Review Board
ITT: Intention to treat
LAZ: Length-for-age z-score
LMICs: Low- and middle-income countries
MUAC: Mid-upper arm circumference
RCT: Randomized controlled trial
RNEC: Rwanda National Ethics Committee
SGA: Small for gestational age
USAID: United States Agency for International Development
WHO: World Health Organization
